# Municipal intermediate care services in Denmark and Norway: a cross-country comparative analysis

**DOI:** 10.1080/02813432.2026.2644949

**Published:** 2026-05-11

**Authors:** Stine Emilie Junker Udesen, Maren Kristine Raknes Sogstad, Marijke Veenstra, Annmarie Touborg Lassen, Marianne Sundlisæter Skinner

**Affiliations:** ^a^ Emergency Medicine Research Unit, University of Southern Denmark, Odense, Denmark; ^b^ Department of Public Health, University of Southern Denmark, Odense, Denmark; ^c^ Centre for Care Research, Norwegian University of Science and Technology–NTNU, Trondheim, Norway; ^d^ Health Service Research Unit (HØKH), Akershus University Hospital, Lørenskog, Norway; ^e^ Department of Emergency Medicine, Odense University Hospital, Odense, Denmark

**Keywords:** Intermediate care, short-term care, municipal, admission avoidance, early discharge, community

## Abstract

**Background:**

Intermediate care is increasingly used to reduce hospital admissions and support early discharge from hospital. In Denmark and Norway, health reforms have shifted responsibilities from hospitals to municipalities, prompting the development of community-based intermediate care services.

**Objective:**

To compare how Danish and Norwegian municipalities have organised and implemented municipal intermediate care services.

**Design:**

A descriptive comparative study based on national legislation, policy documents, research literature and public statistics.

**Results:**

Both countries have established municipal intermediate care structures centred on service types: acute and non-acute intermediate care. In Denmark, acute intermediate care includes statutory acute care teams and optional acute care beds, whereas Norway provides statutory acute care beds only. Acute services in both countries are regulated by national guidelines and aim to prevent unnecessary hospital admissions. Non-acute intermediate care mainly consists of temporary institutional beds used for early-supported discharge, rehabilitation, recovery, nursing home waiting placements and respite care. These services are less strictly regulated and vary between municipalities. In both countries, intermediate care primarily targets older adults with complex health needs exceeding the capacity of ordinary long-term care services. Differences were observed in referral pathways, clinical capacity and the degree of municipal autonomy, reflecting broader contextual conditions such as geography, governance structures and workforce availability.

**Conclusion:**

Although Denmark and Norway pursue similar policy goals, their organisational models for municipal intermediate care differ. Distinguishing between acute and non-acute intermediate care highlights the importance of adapting service models to local structural and demographic conditions and informs policy development and cross-country learning.

## 1. Background

In recent decades, governments across the world have increasingly focused on sustainability in healthcare services and continuity in care to meet the challenges of ageing populations, the increased prevalence of chronic diseases, increased pressure on the need for hospital beds, and high patient expectations [1,2]. Intermediate care comprises services which aim to prevent unnecessary hospital admissions by offering support at home or at a lower level of care after a hospital stay. Core aims of such services are to provide alternatives to hospitalisation, particularly for older people [3–5] and to reduce the risk of poor patient outcomes, for example, functional decline, deterioration of illness, and readmission [6]. Other rationales behind intermediate care are bridge-building between healthcare sectors and improved support in the transition from illness to recovery [4,5]. Generally, to produce intended outcomes, intermediate care services must be tailored to local systems [5].

In Europe, the landscape of intermediate care models is diverse and care can be offered in different settings, including community hospitals, nursing homes, or in people’s own homes [7]. However, all models generally aim to deliver time-limited services for people whose care needs are too complex or intensive to be handled by traditional primary or long-term care providers [4,7]. Although the international literature on intermediate care is growing, systematic cross-country analyses of how such services are organised at the municipal level remain limited. The diversity in the European intermediate care services complicates the evaluation of models across countries [3,4,6]. Much existing research examines specific service models or their clinical effects, whereas broader questions concerning governance structures, municipal responsibilities, and organisational design are underexplored. This underlines an important gap, particularly in the Nordic context, where municipalities hold extensive responsibilities for community-based healthcare.

In the last decade, two Scandinavian countries have introduced policies which drive the implementation of acute municipal intermediate care schemes on a national scale. Since 2016, Norwegian municipalities have been obligated to provide acute care beds [8]. Two years later, in 2018, it also became mandatory for all Danish municipalities to provide acute care, by means of either acute care beds or teams [9]. Later, in 2023, the acute care teams became mandatory [9]. This development was driven by population ageing and resource shortages in the healthcare sector and the resulting demand for improved service integration [3,10–12]. Taken together, these reforms underscore the need for comparative research that examines how countries facing similar systemic challenges have organised municipal intermediate care within different governance and organisational frameworks.

The aim of this study is therefore to analyse how Denmark and Norway have organised municipal intermediate care services in response to recent health reforms, and to contribute to conceptual clarification of intermediate care. The focus on Denmark and Norway is based on their structural comparability as well as their differing approaches to the implementation of acute intermediate care services.

### 1.1. The Danish and Norwegian healthcare systems

As Nordic welfare states, both countries have universal, tax-funded healthcare systems with comparable institutional frameworks, making them well-suited for policy comparison. Both healthcare systems are divided into primary (including long-term care) and secondary healthcare services and organised into three levels: state, regional, and local [1,13]. In both countries, the national (state) level defines the framework of the healthcare system through laws, policies, budgets, national guidelines, and healthcare monitoring. In Norway, specialist health services are administered by four Regional Health Authorities (RHAs), but the responsibility for specialist care lies with the state. In Denmark, the regional level consists of five regions, which are responsible for general practitioners (GPs), hospitals, specialist care, and specialised residential care. At the local level in both countries, the municipalities are responsible for long-term care (nursing homes and home care) and some primary healthcare services, including prevention, health promotion, and rehabilitation [1]. In both countries, GPs act as the gatekeepers to specialised care and are self-employed but embedded in the public system [1]. Danish and Norwegian home nursing is free of charge, but residents living in nursing homes and other long-term care facilities must pay for rent and different services. Danish home care (practical help) is free of charge [1], whereas Norwegian home care is based on cost-sharing [14]. In both countries, these long-term care services are staffed by a mix of nurses, nursing assistants, and unskilled staff [1,15].

### 1.2. Recent developments in long-term care in Denmark and Norway

Ageing in place policies have been advocated in Denmark and Norway for decades, and national health policies highlight the challenges of population ageing for sustainable healthcare and the importance of creating better transitions within and between sectors [15,16]. In both countries, there have been major health system reforms. The Danish government’s Structural Reform from 2007 [17] and the Norwegian government’s Coordination Reform from 2012 [18] set a new course for the two countries’ healthcare systems. With these reforms, responsibility for complex health tasks shifted from specialist services to municipalities, aiming to strengthen prevention, early detection, and community-based treatment and follow-up [18,19]. Ageing in place—living at home with support—has become the norm for older people with care needs [15], and institutional care has become increasingly specialised in recent years [10]. It is in this landscape that municipal intermediate care services have materialised in Denmark and Norway to meet the rising demand for time-limited care and to reduce pressure on hospitals.

**Figure 1.**
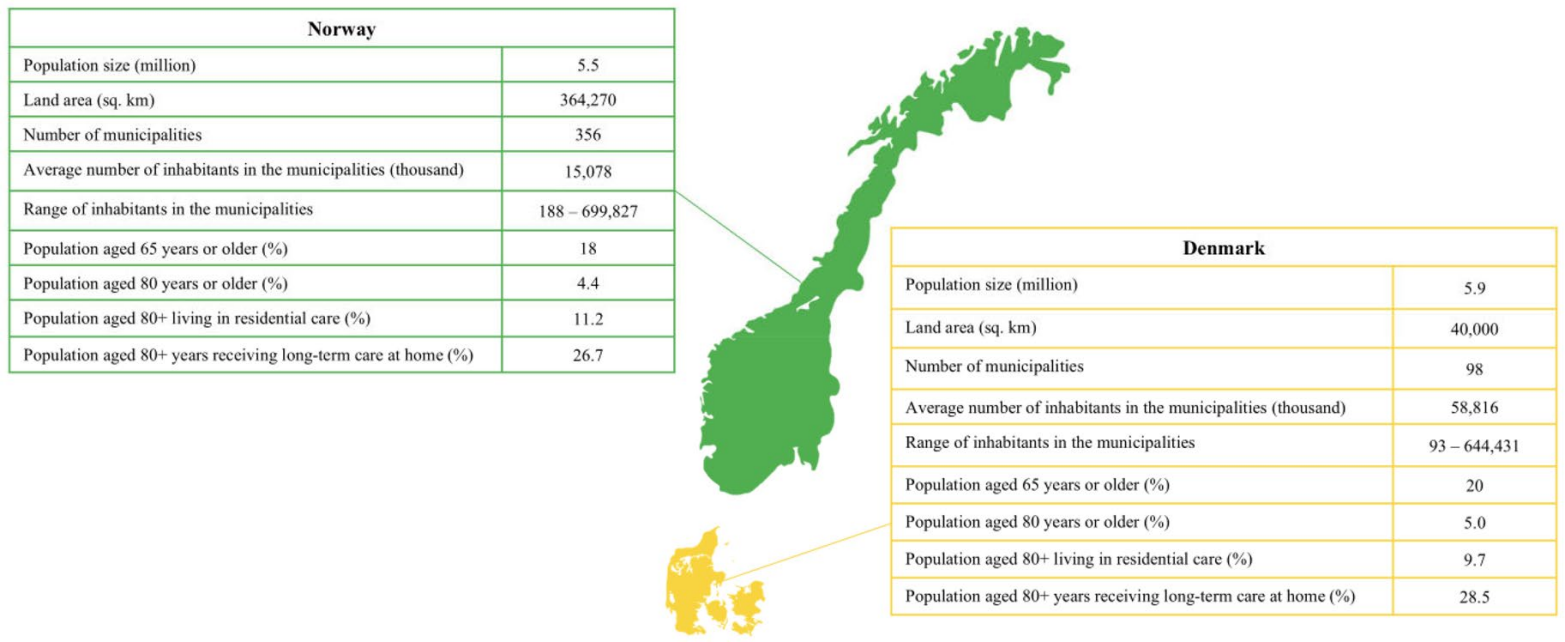
Key demographic and long-term care indicators in Denmark and Norway. Note to Figure 1—references: Population size [79]; land area [80]; number of municipalities and average number of inhabitants [1]; range of inhabitants [81,82]; population aged 65 years or older [83]; population aged 80 years or older [81,82]; population aged 80+ years living in residential care or receiving long-term care at home [22].

As illustrated in Figure 1, Denmark’s and Norway’s population sizes are comparable, but Norway’s vast land area, diverse geography, and high number of small municipalities make the two countries’ contexts and premises for organising their health and long-term care services very different. Denmark’s population is slightly older than Norway’s. In 2022, Denmark had 44,088 long-term nursing home beds [20], equivalent to covering 15.1% of the population aged 80 years and older, while Norway had 29,004 long-term nursing homes beds [21], equivalent to covering 12.1% of the population aged 80 years and older. Based on 2021 statistics from the Organisation for Economic Co-operation and Development (OECD), 9.7% of the Danish population above 80 years lived in residential care, and 28.5% received long-term care services at home [22]. Based on the same statistics, 11.2% of the Norwegian population above 80 years lived in residential care and 26.7% received long-term care services at home [22].

## 2. Methods

A descriptive–comparative design was chosen to map and analyse national models of municipal intermediate care in Denmark and Norway. This approach was appropriate given the study’s aim of comparing how two structurally similar welfare states have organised intermediate care, and because it allows synthesis of heterogeneous data sources, including legislation, national guidelines, statistical reports, and research literature.

A preliminary literature search was conducted to identify existing knowledge and to inform the construction of the analytical framework. The search was carried out in Scopus, selected for its broad coverage of health policy, social care, and organisational research, which were central to the study. Although databases such as PubMed offer strong biomedical coverage, they capture fewer municipal- and policy-level studies, and they were therefore not used. The search covered publications from January 2010 to May 2024, using the following string: [municipal* OR communit* W/2 (intermediate OR unit* OR ward* OR team* OR nurs* OR in-patient OR rehabilita* OR recover* OR post-hospital OR next-kin OR palliati* OR acute OR healthcare)] AND (Denmark OR Danish OR Norway OR Norwegian). Inclusion criteria were studies on municipal or community-based intermediate care, published in English, Danish, or Norwegian; commentaries and studies without organisational relevance were excluded. Of the 605 studies identified, none provided a national overview of either country or a cross-country comparison of Denmark and Norway.

Insights from the search guided the construction of the analytical framework, which drew on two studies: First, the international Delphi study by Sezgin and colleagues, which identified consensus-based characteristics of intermediate care models across 13 countries, including defining features, purposes, target groups, care models, and organisational structures [23]. Second, the comparative framework developed by Steele Gray and colleagues, which emphasises the importance of analysing how programme structures interact with broader policy and system contexts in cross-country comparisons [24].

Building on these foundations, we adapted the analytical framework to align with the study’s emphasis on municipal responsibilities and the governance arrangements that structure intermediate care in Denmark and Norway. Rather than treating programme structures and policy contexts as separate analytical domains, as in Steele Gray and colleagues’ framework [24], we integrated relevant contextual factors into the interpretation of findings across the analytical dimensions, for example, as municipal autonomy, regulatory requirements, geography, and workforce availability.

The final analytical framework (Table 1) comprises five dimensions: definitions, purposes, target populations, care models, and organisation of care, and guided the extraction and synthesis of data.

**Table 1.** Analytical framework to identify key characteristics of intermediate care services.

Main categories	Subcategories
1. Definitions	Legislation Guidelines or requirements Setting
2. Purposes	Purpose Outcomes Duration of care
3. Target populations	Patient groups Referral causes/diagnoses
4. Care models	Entry point Medical responsibility Equipment Communication and coordination
5. Organisation of care	Staffing and competencies Capacity Funding

*Source:* Inspired by Sezgin et al. [23] and Steele et al. [24].

## 3. Results

Based on the analytical framework, the findings are elaborated on in the following five sections: (1) definitions, (2) purposes, (3) target populations, (4) care models, and (5) organisation of care. A comprehensive overview of the key characteristics of municipal intermediate care services in Denmark and Norway is provided in a table in Appendix A, while the main cross-country differences are summarised in Tables 2 and 3.

**Table 2.** Key differences in acute intermediate care (Denmark *vs.* Norway).

Theme	Denmark	Norway
Definition and legal basis	National recommendations since 2014; statutory requirements since 2018; mandatory acute care teams (from 2023); acute beds optional.	Introduced by 2012 Coordination Reform; acute beds mandatory since 2016; expanded to psychiatric and substance-use patients from 2017.
Organisation	Two types: acute care teams (home-based, including hospital-at-home services) + acute care beds (facility-based).	One type: acute care beds only, always facility-based; no hospital-at-home.
Purpose	Prevent unnecessary hospital admissions *via* early diagnosis/timely intervention; support early discharge.	Prevent admissions by providing locally accessible acute care within municipal settings.
Length of stay	Short stays of a few days.	Same.
Target population	Adults with acute somatic conditions or unclear symptoms; mostly older adults. Psychiatric patients are not formally included.	Similar somatic focus; also, psychiatric and substance-use patients since 2017.
Referral pathways	Very broad: GPs, out-of-hours GPs, municipal staff, hospitals, ambulance staff, emergency dispatch.	Supposed to be only primary care physicians.
Medical responsibility	Always with the referring physician or hospital department.	Referring physician until municipal physician assesses patient.
Clinical capabilities	Clear, detailed national specifications incl. bedside diagnostics and equipment requirements.	Less detailed requirements.
Care model	Proactive, preventive, cross-sectoral; strong support to municipal staff; early intervention focus.	Bed-based, reactive, emphasising local accessibility.
User fees	No payment.	Same.

**Table 3.** Key differences in non-acute intermediate care (Denmark *vs.* Norway).

Theme	Denmark	Norway
Definition and legal basis	‘Temporary stays’, ‘short-term stays’. Optional service (but provided in all municipalities). No national regulation but rehabilitation stays require therapeutic competencies.	Same terminology. Mandatory service. Guidelines for rehabilitation and palliative care; otherwise, no overarching regulatory framework.
Organisation	Mostly located in nursing homes or special municipal facilities; co-location with acute beds less common.	Same locations; frequent co-location with acute beds.
Purpose	Short-term treatment, rehabilitation, observation, recovery, respite care; avoid prolonged hospital stays.	Same purposes; palliative care explicitly included.
Length of stay	Typically, one week to a few months.	Same.
Target population	Broad group with short-term needs.	Same.
Referral pathways	Managed by municipal service allocators.	Same.
Medical responsibility	Usually GPs; after hospital discharge, hospitals retain responsibility for first 72 h.	Varies: patient’s GP or a municipal or nursing home physicians depending on municipal organisation.
Clinical capabilities	Less advanced than acute care; no statutory requirements for equipment.	Same; palliative and rehabilitation capabilities defined.
User fees	Small fee charged.	Small fee charged.
Capacity	3230 beds (including acute care beds)	10,061 beds (including acute care beds).

### 3.1. Definitions

**Figure 2.**
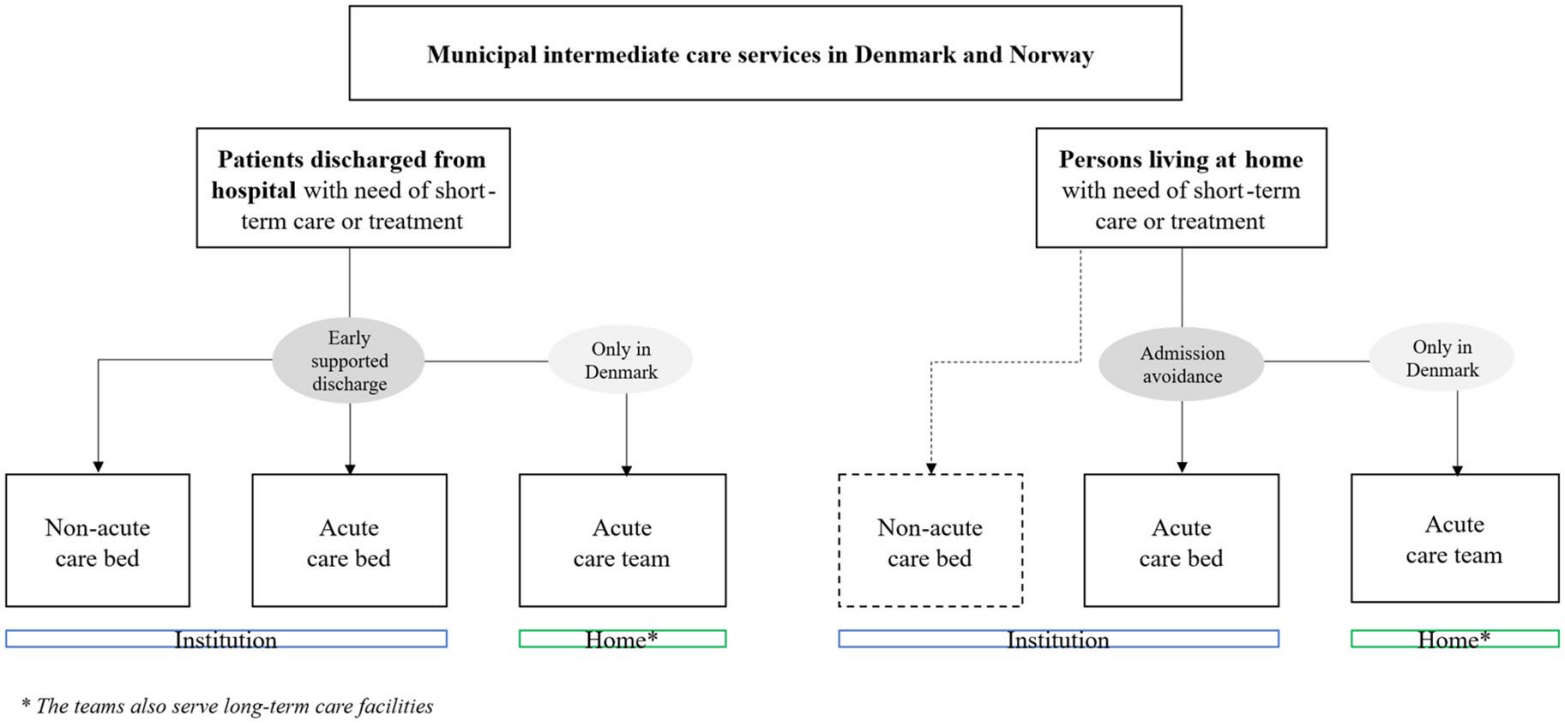
Flowchart of intermediate care services in Denmark and Norway.

Municipal intermediate care in Denmark and Norway can be categorised into two main types: acute and non-acute services (Figure 2).

#### 3.1.1. Definition of acute municipal intermediate care

As mentioned in the background, the introduction of the acute municipal intermediate care service was based on national guidelines in both countries [8,9]. In Denmark, recommendations care were first issued in 2014 [25], and since 2018, municipalities have been required to provide acute intermediate care to adult people with somatic diseases, either through acute care teams or acute care beds [9]. In 2023, Danish acute care teams became statutory, while acute care beds remained optional [9]. In Norway, acute care beds were introduced through the 2012 Coordination Reform [18]. Some municipalities had established similar beds earlier [18,26]. Since 2016, all Norwegian municipalities must offer acute care beds for adults with somatic diseases or deterioration of chronic conditions manageable in the community setting [8].

In Denmark, acute municipal intermediate care consists of statutory acute care teams providing care in patients’ homes and optional acute care beds providing care at nursing homes or at other care facilities. In contrast, Norwegian acute municipal intermediate care consists only of statutory acute care beds located in nursing homes or other care facilities. The labels that have been used in literature for the Danish model include ‘acute community healthcare services’ [27] and ‘municipal acute care teams’ [28,29] or ‘acute teams’ [30]. In the literature, the Norwegian acute care beds are variously referred to as ‘municipal in-patient acute care’ (MIPAC) [31], ‘municipal acute units’ [32], and ‘municipal acute wards’ [33].

#### 3.1.2. Definition of non-acute municipal intermediate care

The terminology applied to non-acute municipal intermediate care is similar in the two countries, where ‘temporary stays’ and ‘short-term stays’ are most widely used in both countries [34–36]. In both countries, these non-acute care beds are placed in nursing homes or other care facilities. Danish non-acute care beds are not subject to national statutory requirements; however, rehabilitation stays require staff to possess therapeutic competencies [39]. In Norway, specific guidelines define the purpose and scope of palliative and rehabilitation care [40,41], but no unified regulatory framework governs the broader non-acute municipal intermediate care category. The Norwegian municipalities are obligated to offer non-acute intermediate care but free to decide how to organise the services [42,43]. All Danish municipalities offer non-acute beds, but it is optional, and the capabilities and capacity of the services differs [12].

### 3.2. Purposes

Intermediate care services in Denmark and Norway serve broadly similar functions, aiming to bridge the gap between hospital and home by preventing unnecessary hospital admissions and supporting early hospital discharge.

#### 3.2.1. Purpose of acute municipal intermediate care

The overall purpose of acute municipal intermediate care services in both countries is broadly comparable, although operationalised in different ways (Figure 2). In Denmark, acute intermediate care is intended to prevent unnecessary hospital admissions through early diagnosis and timely intervention, and to support early discharge from hospital for people with complex health needs (illustrated in Figure 2) [9]. In Norway, the purpose is similar to prevent unnecessary hospital admissions [8]. In both countries, the acute care beds are designed for short stays, typically lasting only a few days [8,9].

#### 3.2.2. Purpose of non-acute municipal intermediate care

The purposes of non-acute care beds also show substantial similarities across Denmark and Norway. The beds are used to provide short-term care for treatment, rehabilitation, recovery, clinical assessment, and observation [35–38]. In practice, these beds play a central role in facilitating early discharge from hospital. In both countries, non-acute beds are used not only for intermediate care but also for respite care and as waiting positions for long-term nursing home placement. In both countries, placement in non-acute beds is based on an individual needs assessment and commonly lasts from one week to a few months.

### 3.3. Target populations

The main target population for municipal intermediate care in Denmark and Norway is people requiring short-term care before, instead of, or after hospitalisation. These individuals typically have acute or complex health needs that exceed the support available through ordinary long-term care, often due to physical illness, functional decline, or cognitive impairment.

#### 3.3.1. Target population of acute municipal intermediate care

Acute municipal intermediate care targets adults with clarified acute somatic diseases or unclarified somatic symptoms requiring further observation or diagnostic clarification [8,9]. Examples of referral causes are listed in Appendix Table A1. For example, national statistics show that the most common admission diagnoses in Norwegian acute care beds include musculoskeletal conditions (19%), respiratory difficulties (18%), and non-specific symptoms (18%) [44]. Comparable national-level data are not available for Denmark. In Norway, the target group has included patients with psychiatric conditions and substance use problems since 2017 [8]. Some Danish municipalities also have local ambulant teams or acute care beds targeted at people with psychiatric health issues, but they are not mentioned as a target group in the Danish guidelines for acute care beds [9].

In both countries, acute services predominantly serve older adults. Norwegian data indicate that almost half of acute care bed patients are 80 years or older [44], while Danish municipal data show a median age of 80 among patients receiving care from acute teams [28,45].

#### 3.3.2. Target population for non-acute municipal intermediate care

The non-acute target groups are broad and largely consistent across the two countries. In Denmark, non-acute beds are commonly used for observation and assessment, rehabilitation and recovery, respite care, and as waiting positions for long-term care [37]. In Norway, they are similarly used for assessment and treatment, rehabilitation, respite care, and palliative care [10,40,42,43,46]. Although both countries assign municipalities responsibility for end-of-life care, Denmark additionally provides specialised hospice services. Nevertheless, palliative care may also be delivered within Danish non-acute beds when required. Admission to non-acute beds occurs for multiple reasons, but a central function is to prevent unnecessarily prolonged hospital stays by providing a safe transitional setting. A Danish study found that 70% of patients admitted to non-acute beds had recently been discharged from hospital, that 73% subsequently returned to their own home, and that 16% died within 30 days [34]. A Norwegian study similarly reported that 80% of patients admitted to non-acute beds following hospitalisation were able to return home, while 11% died within six months [47]. This underscores the central role of non-acute beds in facilitating early supported discharge, providing short-term stabilisation and rehabilitation for older adults with complex health needs.

### 3.4. Care models

Referral pathways, medical responsibility, and the clinical capabilities embedded in municipal intermediate care services vary across Denmark and Norway.

#### 3.4.1. Care models for acute municipal intermediate care

Acute municipal intermediate care services in both countries are designed with more advanced capabilities than non-acute beds. The Danish guideline requires acute services to have access to paraclinical bedside equipment, the ability to provide intravenous treatment, and staff with appropriate clinical competencies [9]. In Denmark, acute care teams also deliver hospital-at-home services in collaboration with hospitals, which demands advanced nursing skills and cross-sectoral agreements between hospitals and municipalities [9]. These hospital-at-home services are not a part of the Norwegian acute care bed model, which remains facility-based and primarily focused on preventing hospital admissions. The Norwegian guideline likewise require staff to have appropriate clinical competencies but do not specify requirements for bedside equipment [8].

The Danish guideline is also more detailed regarding specification of medical responsibility compared to the Norwegian guideline [8,9]. In Denmark, referrals to acute care teams may originate from GPs, out-of-hours GPs, hospital physicians, municipal healthcare staff, ambulance staff, or the emergency medical dispatch centre [9]. This wider referral model allows earlier intervention and reflects greater cross-sectoral integration than in Norway. Admission to the Danish acute care beds requires a referral from a physician when treatment is initiated, and in such cases, the referring physician has the medical responsibility [9]. The referring hospital department or physician is medically responsible for the patient [9]. Statistics for selected Danish municipalities show that the majority of the referrals to acute care beds and acute care teams come from GPs and municipal healthcare staff [27,28,48,49]. Acute care teams in Denmark also provide an established support function for municipal staff, offering timely intervention, which may prevent hospital admissions [3,45]. This proactive, preventive orientation distinguishes the Danish model from Norway’s more facility-based acute care beds.

In Norway, admission to acute care beds requires referral from a physician. Medical responsibility typically remains with the referring physician until the patient is assessed by the municipal physician, which may take several hours or longer if admission occurs outside normal working hours [50]. In large Norwegian acute care bed units with a 24-h physician service, the handover of medical responsibility is instant upon admission [51]. The majority of referrals come from out-of-hours GP clinics (60%), followed by daytime GPs (21%) and hospital physicians (16%) [44]. Although referrals from hospitals were not originally intended, they occur in practice [44] and may reflect organisational contingencies or regional variation [48]. In this context, Norwegian guidelines note that some patients may require specialist-level diagnostic evaluation before being admitted to acute care beds [52]. The majority of the patients are referred in out-of-hours (70%) and are directly transferred to the acute care bed from their homes (85%) [44]. The majority of patients admitted to the acute care beds are discharged to their homes (64%), and the remainder are discharged to long-term care facilities or non-acute care beds (18%), to hospital (14%), or to other facilities (3%) [44].

#### 3.4.2. Care models for non-acute municipal intermediate care

The process of admission to non-acute care beds is similar across the two countries: municipal service allocators assess needs and allocate stays at non-acute municipal care facilities. For the Danish non-acute beds, the individual patient’s GP usually has the medical responsibility, but a new effort has been implemented for patients admitted from hospitals to ensure continuity across sectors. Today, the referring hospital ward has the medical responsibility for 72 h after patients are admitted to the Danish non-acute care beds [53]. For the Norwegian non-acute care beds, the medical responsibility varies across municipalities and depends on the organisational structure and agreements with the local GPs. Some municipalities have nursing home GPs who have the medical responsibility for the nursing home residents as well as for the patients admitted to non-acute (and acute) beds in the nursing home [50].

### 3.5. Organisation of care

The organisation of municipal intermediate care services differs significantly between Denmark and Norway, reflecting regulatory, structural, and geographical factors.

Denmark has in total 3230 municipal intermediate care beds [20], whereas Norway has 10,061 such beds [21], including 688 acute care beds [54]. In 2022, the number of admissions to Norwegian acute care beds was nearly 40,000 [54]. Comparable national statistics on Danish acute care admissions are not available.

#### 3.5.1. Organisation of care for acute municipal intermediate care

There is considerable variation in the organisation of Danish acute municipal intermediate care services [27]. Some municipalities operate independent acute care teams, while others integrate them into existing nursing home units [12,37]. Personnel typically include registered nurses with acute or hospital experience [12]. By 2020, 93% of municipalities had acute teams, 56% provided acute beds, and 48% offered both [27]. Smaller municipalities are encouraged to establish intermunicipal collaboration [9].

In Norway, acute care beds vary widely across municipalities depending on local service structures, geography, and demographics [31,54–56]. Many small municipalities operate only one or two beds, often located in nursing homes, and frequently collaborate with neighbouring municipalities to fulfil statutory requirements [31,54–56]. In 2019, 67% of the municipalities participated in intermunicipal collaborations [54]. Some municipalities co-locate acute care beds with out-of-hours GPs [44]. Staff employed in the Norwegian acute care bed service must have competencies to provide care for both psychiatric and somatic patients. Furthermore, many Norwegian acute care beds are co-located with non-acute care beds, which requires other nursing competencies. Sufficient nursing competencies are found to be a challenge for the Norwegian model of acute care beds and particularly for the acute care beds co-located in nursing homes or other long-term care facilities [31]. Nursing competencies have not been investigated for municipal acute care services in Denmark, but as yet, recruitment does not seem to have been a problem for implementing the services [27].

#### 3.5.2. Organisation of care for non-acute municipal intermediate care

The organisation of non-acute beds is relatively similar in both countries. These beds are typically located in nursing homes or special municipal facilities, and patients pay a small user fee [10,37,39,57–60]. In both countries, there is no patient payment for acute municipal intermediate care services [37,39,57–59].

## 4. Discussion

This study has shown that Denmark and Norway have developed broadly similar municipal intermediate care systems, structured around acute and non-acute services, yet they have employed different frameworks and organisational solutions. Both systems aim to prevent unnecessary hospital admissions and support early discharge for people with complex health needs. Particularly targeted are older adults who are vulnerable to adverse health outcomes, for example, delirium, functional decline, and hospital-acquired infections [61–63]. Over the past two decades, both countries have expanded their non-acute bed capacity to meet rising demand for specialised, short-term community-based care [37,64]. Despite similar policy goals, the two welfare states have adopted distinct organisational pathways. Denmark requires municipalities to provide acute care teams, with acute care beds remaining optional, whereas Norway requires municipalities to provide acute care beds only.

These divergent pathways may reflect broader contextual differences. Our findings underline that Norwegian municipalities have greater scope to interpret and adapt national guidelines, as national guidance is less prescriptive regarding required equipment, clinical capabilities, and medical responsibility than in Denmark [8,9]. This flexibility allows municipalities to shape acute care beds to local needs but also contributes to substantial variation. Such variation has implications for equity, as patients’ access to diagnostic equipment, hospital-at-home services, clinical follow-up, and timely intervention may depend on municipal capacity. Geographical factors further reinforce these disparities. Norwegian studies have found that long travel distances, the size of the municipalities, dispersed settlement patterns, and challenging terrain affect physician availability and limit opportunities for intermunicipal collaboration [55,65]. In contrast, Denmark’s compact geography and more centralised governance structure may facilitate greater standardisation, in line with a study showing stronger national regulatory control in Denmark compared to Norway [66]. This increased standardisation may strengthen system sustainability by promoting a more balanced distribution of responsibilities and competencies across municipalities. On the other hand, individualisation of care is essential to ensure that the patient’s condition, circumstances, and preferences are addressed, even within a standardised care framework [67].

Empirically, the study contributes a systematic mapping of Danish and Norwegian municipal intermediate care services—an overview that has thus so far been absent from the literature. By distinguishing between acute and non-acute services and outlining their definitions, purposes, target populations, care models, and organisation of care, the study clarifies the role of these increasingly important community-based services situated between hospital and long-term care. Acute municipal intermediate care in both countries mainly function as ‘admission-avoidance’ but can also facilitate ‘early supported discharge’—established categories in research [5,68,69]. In Denmark, acute teams also provide a proactive support function to other municipal healthcare staff, enabling timely intervention and early detection of deteriorating conditions. A qualitative study highlighted that this feature was experienced as highly valuable in practice for municipal healthcare staff [29].

Moreover, Denmark operates with a wider referral pathway: patients may be referred to acute care teams not only by physicians, but also by municipal healthcare staff, emergency services, and out-of-hours services. This wider referral base reflects a higher degree of cross-sectorial integration and may contribute to timelier interventions and increase the possibilities of preventing unnecessary hospital admissions compared to the Norwegian model. This may also reduce the risk of functional decline or hospital-acquired complications by avoiding unnecessary hospital admissions. A Danish register-based study evaluating a local acute care team demonstrated reductions in acute ambulance transfers and hospital admissions for selected diagnoses [45], indicating that such teams hold potential to prevent acute hospital care. In Norway, 16% of the acute care bed admissions are initiated by hospital physicians, even though the model was intended to be primary care led [8,44]. This fact illustrates that hospital services play a more prominent gatekeeping role than originally planned, potentially undermining continuity of care and blurring the boundary between secondary and municipal responsibility. It may also indicate that some municipalities lack sufficient diagnostic capabilities or competencies to safely assess patients before admission to an acute care bed. A qualitative study also found that healthcare professionals reported frequent disagreements about which patients were appropriate for acute care beds, highlighting uncertainty in clinical assessment and limited medical capacity within local units [70]. In contrast, a register-based study found that the introduction of acute care beds was associated with reduction in both mortality rates and hospital readmission rates among older people [71], indicating positive impact on public health outcomes.

Conceptually, the findings underscore that municipal intermediate care in general should not be viewed as a fixed set of services but rather as a flexible organisational domain spanning preventative, acute, rehabilitative, and supportive functions. Although the distinction between ‘admission avoidance’ and ‘early supported discharge’ is useful [5,68,69], our analysis shows that services rarely operate within a single functional category and often fulfil multiple purposes depending on needs and local organisation. For example, the non-acute beds in both countries provide multiple functions beyond municipal intermediate care, including respite care, palliative care, and waiting positions for long-term nursing home allocation. This demonstrates functional flexibility despite differing legislative frameworks.

Overall, the empirical and conceptual contributions of this study strengthen the understanding of municipal intermediate care as a central component of contemporary health and long-term care systems. The analysis also shows that intermediate care is not simply a technical response to hospital pressures, but a strategic policy choice concerning how responsibilities and capacities are distributed across sectors and levels of governance.

### 4.1. Strengths and limitations

A strength of this study is the systematic, structured mapping of municipal intermediate care services in Denmark and Norway. By drawing on national legislation, guidelines, reports, public data, and published research, the study provides a level of transparency and comparability that is often lacking in this field. This approach allowed us to identify key characteristics of acute and non-acute intermediate care across the two systems, as viewed from a national health care system perspective.

Several limitations must also be acknowledged. First, the exclusive use of Scopus is acknowledged as a limitation. Second, the analysis relies on public available national data, which may differ in scope, detail, and registration practices between the two countries. Such variation may influence the comparability of specific indicators across Denmark and Norway. To minimise these limitations, we report data with as much accuracy and transparency as possible. Fragmented data on intermediate care services is also a challenge for the UK countries [72], underscoring that data constraints are a general issue in this field. Furthermore, it is important to re-emphasise that the Danish and Norwegian non-acute care beds are also used for non-intermediate care purposes. We have been unable to find or provide statistics that reveal how widespread these uses of the beds are; thus, we may have slightly overestimated the capacity of non-acute beds.

Another central limitation concerns the study’s focus on only two countries. This choice reflects practical considerations—most notably language constraints and available resources—but also has a deliberate conceptual rationale. Denmark and Norway are the two Nordic countries that have implemented the most comparable reforms for municipal acute intermediate care. Moreover, their broadly similar healthcare structures and relatively strong national regulatory governance make them particularly suitable for detailed policy comparison, in contrast to, for example, Nordic neighbors’ Finland and Sweden, where municipal responsibilities and national-level governance differ more substantially [1].

### 4.2. Policy and future research

The high demand for intermediate care services is shown by the emerging service landscape of such services across Europe. This development, combined with the challenges associated with ageing populations, highlights the need for greater policy attention and more research within this field. According to the OECD, it is critical to obtain stronger evidence on how care is delivered across service pathways and to identify policies to improve integrated care [2]. A 2025 OECD report also emphasised a broader European and Nordic shift towards community-based intermediate care models, which have been shown to reduce hospital admissions and support ageing at home policies [73]. In this context, the findings of the present study provide insights into intermediate care pathways and policies in Denmark and Norway. The mapped differences and similarities between the countries may be valuable when assessing the feasibility of transferring services from one municipal context to another, or from one national context to another. The findings also highlight important considerations for scalability and transferability. While certain organisational elements—such as Denmark’s wider referral pathways or Norway’s statutory acute care beds—may theoretically be diffused to and adapted across other healthcare systems, their fit and feasibility is shaped by contextual factors including geography, GP availability, workforce availability, regulatory frameworks, and municipal capacity. These conditions may limit the straightforward transfer of service models, suggesting that any scaling efforts would require careful contextual adaptation and translation rather than direct replication [74,75].

Furthermore, the Danish and Norwegian governments have high expectations for municipal acute intermediate care, and these services are positioned as a part of the emergency chain [8,78]. This makes it important to increase knowledge within this field. For example, there is a need for more knowledge about the competencies required to operate municipal intermediate care services effectively. Norwegian studies have examined clinical competencies within acute care beds [31,76], whereas similar research is largely absent in Denmark, highlighting an important knowledge gap for future investigation. Additionally, it is essential to ensure that intermediate care represents the best option for the individual patient and that the physical environment, competencies, and overarching policies align with patient needs. Future research should prioritise this focus and provide transparency in descriptions of municipal intermediate care services to enable robust comparisons and informed decision-making. This also underscores the importance of obtaining more evidence about various forms of intermediate care services.

Lastly, the findings are highly relevant given the new Danish healthcare reform planned for implementation in 2027 [77]. Under this reform, responsibility for several tasks will be transitioned from municipalities to regions, including acute care teams, acute care beds, and nearly 70% of non-acute care beds [77]. This transfer requires well-informed knowledge of existing intermediate care services, making this study particularly relevant and timely for Danish decision-makers. Furthermore, researchers and stakeholders in other countries may find the analytic framework useful for identifying and comparing key characteristics of intermediate care services and service pathways. An enhanced understanding of municipal intermediate care can support efforts to target and expand types of services and ensure their sustainability within the broader health and long-term care systems.

## 5. Conclusion

This study compared Danish and Norwegian municipal intermediate care policies and showed that both countries organise services around acute and non-acute pathways aimed primarily at avoiding unnecessary hospital admissions and early supported hospital discharge. Despite these similarities, their organisational models differ: In acute municipal intermediate care services, Denmark combines acute care teams with optional acute beds, enabling a proactive and flexible approach, whereas Norway relies solely on statutory acute care beds and allows greater municipal autonomy in design and organisation of services, reflecting substantial geographic and demographic variation across municipalities.

Non-acute services in both countries support early supported discharge and provide rehabilitation and recovery functions but remain heterogeneous and weakly regulated. Their organisational flexibility makes them a central component of the intermediate care continuum, functioning as the interface between hospital services, acute municipal intermediate care, and long-term care.

Overall, the findings demonstrate that intermediate care must be adapted to local structural and governance conditions rather than implemented as a uniform model. These insights are relevant for ongoing policy development in Denmark and Norway, for policymakers in other countries seeking cross-national learning, and for local, national, and international stakeholders and researchers working to strengthen community-based care through a more integrated intermediate care continuum.

## Appendix A

**Table A1.** Key characteristics of intermediate care services in Denmark and Norway.

Main categories	Subcategories	Denmark		Norway	
		Non-acute	Acute	Non-acute	Acute
1. Definitions	- Legislation - Guidelines or requirements	- Based the Consolidation Act on Social Services, §84.2 [59]. - No national guidelines exist. The municipalities are not obligated to provide non-acute intermediate care [32].	- Based on the Health Care Act, §138 [84]. - National recommendations for the implementation of acute care beds and acute care teams were introduced in 2014 [25]. National guidelines were published by the Danish Health Authority in 2018 and 2023 defining target groups, collaboration, staffing, medical responsibility, equipment, organisation, competencies, documentation, monitoring, etc. [9].	- Based on the Act relating to Municipal Health and Care Services, § 3-2 [85]. - No national guidelines exist. The municipalities are obligated to provide non-acute intermediate care [42,43]. Specific guidelines define palliative and rehabilitation care [40,41].	- Based on the Municipal Health and Care Services Act, § 3–5 [85]. - The first acute care beds were established in 2012, and since 2016, it has been mandatory for all Norwegian municipalities to offer acute care beds [8]. National guidelines were published by the Norwegian Directorate of Health in 2012, 2013, 2014 and 2016, which defined the target groups, collaboration, organisation, staff, competencies and documentation [8].
2. Purposes	- Purpose - Outcomes - Duration of care	- The purpose is broad and mainly for older people who either need short-term care for treatment, care or preventative purposes or who are not safe staying at home [37]. - The outcome is dependent on the purpose of the stay, but it is often recovery from surgery, injuries or disease after hospital admission or avoidance of hospital (re)admission. - From weeks to a few months [33].	- The purpose is to avoid hospitalisation through early diagnosis and timely treatment [9]. Supports early hospital discharge for patients with complex health or care needs [9]. - The outcome is avoidance of hospital (re)admissions and unnecessary transitions between healthcare sectors by providing treatment in a nearby setting/at home. - Both the acute care beds and acute care teams are for patients who need a few days of care [9].	- The purpose is broad and mainly for older people who would benefit from short-term care or who are not safe staying at home [35,38]. - The outcome is dependent on the purpose of the stay, which is often recovery from surgery, injuries or disease after hospital admissions or avoidance of hospital (re)admission. - Usually two weeks to 60 days [57,58].	- The purpose is to avoid hospitalisations and provide more seamless patient trajectories by offering treatment care in a nearby setting [8]. - The outcome is avoidance of hospital (re)admissions and unnecessary transitions between healthcare sectors by providing treatment in a nearby setting. - The acute care beds are mainly for patients who need a few days of care. Most are discharged within four days [8,54].
3. Target population	- Patient group - Referral causes/diagnosis	- Short-term care for patients (often transitioning from hospital to home) in need of primarily: (1) observation/assessment; (2) recovery/rehabilitation; (3) respite care if next of kin require relief; (4) waiting for long-term care [37,39]. - A wide range of somatic diagnoses/conditions or reduced mental and/or functional capacity.	- Short-term care for patients with: (1) acute clarified somatic diagnosis that can be handled in the community setting or deterioration of chronic condition; (2) unclarified somatic diagnosis who need observation/investigations [9]. Psychiatric patients with somatic needs can also be treated in collaboration with psychiatric health staff [9]. - Referral causes are somatic diseases that can be handled in the community setting (for example, pain or unspecific symptoms, infections, dehydration, breathing difficulties or chronic obstructive pulmonary disease). The acute care team makes clinical nurse assessments at home (for example, for suspected infections) [9,28].	- Short-term care for patients (often transitioning from hospital to home) in need of primarily: (1) assessment/treatment; (2) recovery/rehabilitation; (3) respite care if next of kin require relief; (4) palliative care [10,40,42,43,46]. - A wide range of somatic diagnoses/conditions or reduced mental and/or functional capacity.	- Short-term care for patients with: (1) acute clarified somatic diagnosis or deterioration of chronic condition that can be handled in the community setting; or (2) unclarified somatic diagnosis which needs observation and investigations; (3) psychiatric health or substance abuse issues (since 2017) [8]. - Referral causes are somatic diseases that can be handled in the community setting (for example diagnoses related to the musculoskeletal system, pain or unspecific symptoms, infections, dehydration, breathing difficulties or chronic obstructive pulmonary disease) [8,44,86].
3. Care models	- Entry point - Medical responsibility - Equipment and medication facilities - Communication and coordination	- Municipal service allocators represent the entry point to the non-acute care beds. In some municipalities, physicians can admit patients directly to the non-acute care beds without involving allocators [87]. - No physicians are employed in this service. The patient’s GP most often has the patient responsibility. For patients admitted from hospital, the hospital has the patient responsibility for 72 h after discharge [88]. - Before 2024, it was illegal to store medication without patient identification in Danish municipalities [37]. From 2025, the municipalities can store medication in medication rooms. - There is no access to patient records across sectors. The hospitals only send epicrises to the GPs. The electronic nationwide common medication card ensures that all healthcare professionals have access to the same medication data.	- All physicians, municipal healthcare staff, ambulance staff and the emergency medical dispatch centre can refer patients to the acute care teams. Only physicians can refer patients to the acute care beds, as treatment needs to be initiated [9]. - The referring physicians have the patient responsibility [9]. If an out-of-hours GP refers the patient, the patient’s GP takes over the responsibility in opening hours and vice versa [9]. For patients admitted from hospital, the hospital has the patient responsibility for 72 h after discharge [88]. - Varies. There are national requirements for equipment that must be available, such as bedside equipment for measuring vital parameters, blood sampling and a nebulizer device [9]. Before 2024, it was illegal to store medication without patient identification in Danish municipalities [37]. From 2024, the municipalities can store medication in medication rooms. - There is no access to patient records across sectors. The hospitals only send epicrises to the GPs. The electronic nationwide common medication card ensures that all healthcare professionals have access to the same medication data. Having a direct telephone line from the acute care team or beds to the physician with the patient responsibility is mandatory [9].	- Municipal service allocators represent the entry point to the non-acute care beds [89]. - The patient responsibility varies, as some municipalities have nursing home physicians who serve the beds, whereas others rely on the municipality’s GP services [50]. - Some nursing homes and short-term care facilities store medication in medication rooms, and some also have laboratory facilities. - There is no access to patient records across sectors. The hospitals only send epicrises to the GPs and to nursing home physicians. There is no nationwide common medication card.	- Admissions to the acute care beds require a referral from a primary care physician who has made a clinical assessment of the patient [90]. Some acute care bed units also accept referrals from hospital physicians [91]. - Some municipalities have employed physicians dedicated to the acute care bed unit. In others, it is nursing home physicians or GPs with special agreements who have the medical responsibility during daytime [92]. The out-of-hours GP service often has the patient responsibility after hours [8]. - Some acute care beds have medication rooms and laboratory facilities [93]. There are no national requirements for equipment and utensils [8]. - There is no access to patient records across sectors. The hospitals only send epicrises to the GPs and to nursing home physicians. There is no nationwide common medication card.
4. Organisation of care	- Staffing and competencies - Setting - Capacity - Funding	- Staffed by registered nurses, nursing assistants, other healthcare workers and unskilled staff. In rehabilitation stays, therapeutic competencies must be present [39]. - The non-acute care beds are located in municipal care facilities (either co-located with other services or as an independent unit). - In 2022, 3223 intermediate care beds were registered on the national level (including both non-acute and acute care beds) [20]. - Co-payment for a limited share of services, such as transport, food, rent for bedlinen and towels [37,59].	- The staff must be registered nurses and nursing assistants with specialised competencies [9]. Several of the nurses have hospital experience [12]. The municipalities are responsible for having local procedures and guidelines for competence development [9]. - The mandatory acute care teams provide acute care in patients’ homes. The optional acute care beds are located in municipal care facilities (either co-located with other services or independently). - There are no national statistics on the capacity of the acute care teams. All municipalities must have acute care teams, but acute care beds are optional. - No (co-)payment.	- Staffed by registered nurses, nursing assistants, other healthcare workers and unskilled staff. For palliative and rehabilitation care, specific guidelines for staffing exist [40,41]. - The non-acute care beds are often located in a nursing home [60]. Large and medium-sized municipalities increasingly offer intermediate care in nursing homes devoted to short-term care [10]. - In 2022, 10,061 intermediate care beds were registered on the national level (including both non-acute and acute care beds) [21]. - Patients must pay a patient charge per night. 95% of the cost is paid for by the municipalities [57,58].	- Staffed by registered nurses, nursing assistants, other healthcare workers and unskilled staff [31]. The municipalities are responsible for having local procedures and guidelines for staffing and competence development [92]. - The acute care beds are located in municipal care facilities (either co-located with other services or municipalities or independently). - In 2022, 688 acute care beds were registered in the national statistics, but the real number is likely higher, as only 84% of the municipalities answered the questionnaire [54]. - No (co-)payment.
